# The Riddle of Ball Lightning: A Review

**DOI:** 10.1100/tsw.2006.48

**Published:** 2006-02-26

**Authors:** José Donoso, José Luis Trueba, Antonio F. Rañada

**Affiliations:** ^1^ Departamento de Física Aplicada, ETSI Aeronáuticos, Universidad Politécnica, 28040, Madrid, Spain; ^2^ Departamento de Física Aplicada, Universidad Rey Juan Carlos, 28933 Móstoles, Spain; ,sup>3 Departamento de Física Aplicada III, Universidad Complutense, 28040 Madrid, Spain

**Keywords:** atmospheric electricity, lightning, electrical discharges, plasma physics, electromagnetism

## Abstract

One of the most intriguing and enduring scientific challenges is to find an explanation for ball lightning, the shining fireballs that sometimes appear near lightning strokes. Although many theoretical ideas have been proposed and much experimental work has been performed, there is not yet an accepted explanation of their amazing properties. They are surprisingly stable, lasting up to 10 s, even minutes in some rare cases. By night, their appearance can be spectacular, but their brilliance is just similar to that of a home electric bulb. Most of the time, their motion is smooth and horizontal, but it can also be erratic and chaotic; they can penetrate indoors through window panes. We review here some of the most discussed approaches, including both theoretical models to find an explanation as well as experimental efforts to reproduce them in the laboratory. We distinguish between chemical and physical models, depending on whether their stability is mainly based on their chemical composition or on purely physical phenomena involving electromagnetic fields and plasmas.

## OVERVIEW: THE PHENOMENON

Ball lightning (herein BL) is an intriguing natural phenomenon for which, thus far, there is no scientific explanation; being, probably, the last natural phenomena still unaccounted for in the lower atmosphere. It consists of a flaming ball or fireball, usually bright white, red, orange or yellow, even bluish or greenish, which sometimes appears near the discharge of a normal lightning bolt or, more rarely, in midair coming down, almost vertically, from a cloud. In most cases, it is associated with thunderstorms. Its shape is usually spherical, but it can also be ellipsoidal, toroidal, or tear shaped. In some cases, it is surrounded by an aura or by outgoing narrow, colored streamers. BL tend to move horizontally at a pace often described by witnesses as majestic. Typically, the (optical) diameter is between 10 and 50 cm. The observed distribution of the lifetime has a maximum between 2 and 5 s, and an average value of about 10 s or higher, with reports of more than 1 min in some cases. The fireballs are bright enough to be clearly seen in daylight, the visible output being in the 10–150 W range, similar to that of a home electric bulb. Some balls have appeared within aircraft, travelling from front to rear inside the fuselage along the aisle. There are witnesses who talk about odors similar to those of ozone, burning sulfur, or nitric oxide, and about sounds, mainly hisses, buzzes, or flutters. The majority seem to decay silently, but others do it with an explosion or an implosion. People and animals have been killed or injured by BL, and fires and damage to trees, buildings, cars, or electric equipment have also occurred. This shows that there is something hot inside. In such events, the released energy has been estimated up to about 10 kJ, and more than 1 MJ[1,2,3,4].

The name "ball lightning" is perhaps misleading, since it is not lightning of any kind in the sense of an electric discharge. It is sometimes claimed that, under the name of "ball lightning", several similar, but different, phenomena are included. An example of this is what is sometimes observed in submarines after a short circuit of the batteries: balls of plasma appear between the electrodes and float in the air for several seconds; the current and energy are about 150 kA and 200–400 kJ. According to Stenhoff[4], BL is any bright or flaming ball reported by witnesses that can be neither an ordinary lightning bolt[5] nor a St Elmo's fire, an aurora, or any other well-known phenomenon. He presents an extensive list of phenomena that can be mistaken for BL in his excellent book.

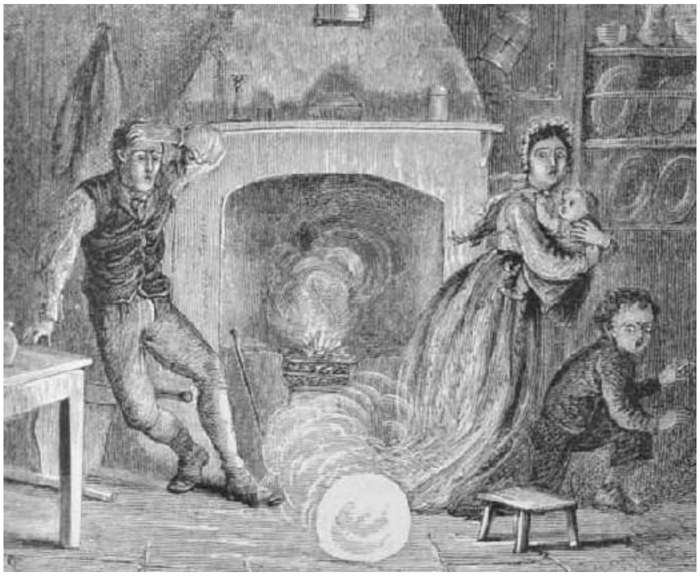

**A**

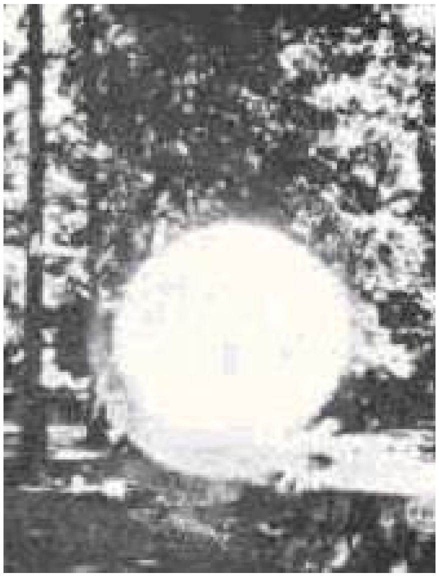

**B**

**FIGURE 1**. (A) "Globe of fire descending in to a room." From G. Hartwig, "The Aerial World," London (1886). (B) Possible ball lightning in Basle, Switzerland (1907).

Although BLs have been seen for several centuries, their scientific study began in the 19th century. It was soon clear that an explanation would not be easy; the main reason being that, as Faraday stated, if they are an electric phenomenon, as it seems most probable, the balls should almost explode instantaneously, in a fraction of a second, and their long lifetime would be impossible to explain. Partly because of this, some researchers attributed them to hallucinations or delusions of the eyewitnesses, but the consensus in favor of a real existing phenomenon is total nowadays. There is not, however, a generally accepted theory that can account for their properties. A great variety of diverse proposed models has, somewhat, contributed to a certain degree of confusion. Since it has been impossible, until now, to produce unequivocal BLs in the laboratory, the research of this phenomenon depends, strongly, on the reports by witnesses who have no scientific training, except for a few exceptions. Since the first collection of such reports by Arago in 1838, there has been an increasing effort to compile and analyze them. Some observation programs include psychologists who are specialists in human perception to ensure the objectivity of the witnesses' accounts.

The reports usually refer to the motion, evolution, and destruction of the fireballs, not to their formation, as it can be easily understood, since they are observed just after their appearance. The search for an explanation of the phenomenon is, thus, difficult, since the appearance and formation of the balls have been rarely described. Some scientists have established contacts with societies of mountaineers, ramblers, or sightseers, asking them to inform about any sight of fireballs and training them beforehand to get a more exact report.

This effort to find statistical objective data bore its fruit, since there are now sure quantitative data that are very useful to test any theoretical model. Considering this, two of the results obtained must be emphasized. First of all, it was found that a lot of characteristics of BL are shared with ordinary lightning, showing standard log-normal distribution for some typical parameters[6], and second, almost all BL events are associated with electric storms. These two facts suggest that BL is an electromagnetic phenomenon. It is true that a few BL events have appeared under clear air conditions without any lightning strikes in the surroundings, which has been used to support nonelectromagnetic models. However, ordinary lightning has also been seen outside stormy situations. This can be explained because some lightning bolts can be produced in a distant storm and be carried by a precursor set of streamers to an unclouded region. In particular, this was the case of the “bolt from the blue” or “Positive Giant”, a lightning strike that hit the ground 20 mi away from the storm in which it was produced, while the associated thunder could only be heard up to about 10 mi. Nevertheless, the possibility of being produced by other effects, such as frictional forces in an earthquake, should not be rejected, as pointed out by some scientists.

Any theoretical model of BL must explain a variety of contradictory properties. According to Singer[7], who steadily stimulated the research on BL, a convincing theory should explain, at least, the three following distinctive characteristics of fireballs: their surprisingly long lifetimes, their floating motion near the ground, and the way in which they disappear. Another characteristic difficult to explain is the ability of BL to pass through window panels or very small holes and narrow slits without, apparently, any change in structure; no model gives a credible explanation of this fact. Another controversial topic is whether the balls are hot or cold, with arguments in favor and against. Some witnesses say that they are cold since a ball passed near their hands without them feeling any warmth, but other balls produced fires and some people were burned, even to the point of needing medical attention for being injured as if by a lightning bolt[8,9].

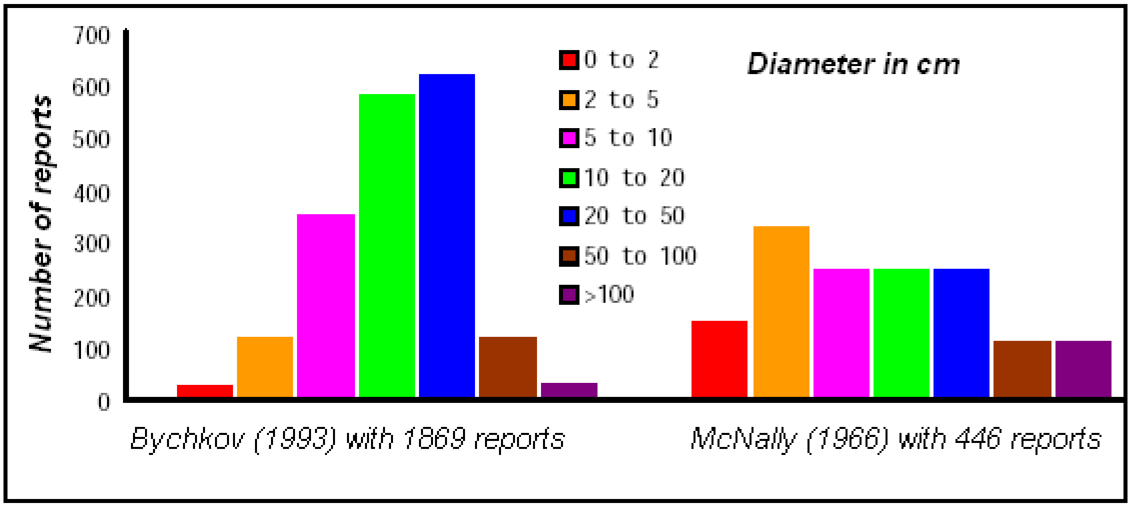

**FIGURE 2**. Distribution frequency for diameter in two surveys; from Stenhoff[4].

The plan of this review is as follows. In the next section, some phenomena frequently mistaken for BL are discussed. Some theoretical models are briefly reviewed after, following the statements summarized in the classification schemes suggested in the third section. We deal with two kinds of models, according to whether they are based on chemical or physical arguments; this is to say, do the arguments given to explain the phenomenon depend, explicitly, on the matter compounds or not? We then consider several experiments that have produced shining structures that resemble the natural BL and follow with a review of some recent models based on plasma physics. The paper ends with a short summary.

## BALL LIGHTNING AND APPARENTLY SIMILAR METEOROLOGICAL PHENOMENA

As reported by Stenhoff, there is a great variety of natural phenomena that can be mistaken for BL; among them, bead lightning and St Elmo's fire are certainly the most similar in their appearance.

### Bead Lightning

Bead lightning**,** also called beaded or chain lightning, consists in an alteration of a normal lightning flash in such a way that it becomes periodically fragmented along its trajectory, looking like a chain of almost spherical luminous sections that tend to slowly dissipate. This phenomenon, usually accepted as a form of lightning, or a way of lightning demise, has been analyzed by J.D. Barry in his interesting book[2]. The occurrence of bead lightning in Nature is more frequent than that of BL, in spite of the fact that the number of reports referring to the former is much lower. This is probably due to the fact that the behavior of bead lightning is more regular than that reported for BL. Among these sets of regularities, it is remarkable that bead lightning is usually associated with cloud-to-cloud linear lightning that split into persistent luminous formations along the initial lightning path lasting about 1–2 s. As indicated by Barry, bead lightning's main characteristic is its dotted appearance that often resembles a quasi-wave structure. The luminous beaded formations seem to decay smoothly and noiselessly, while remaining close to the initial lightning channel. These properties are the main differences between BL and bead lightning phenomena. Although the nature of bead lightning is still under continuous inspection, it seems to be related to lightning channel decay due to periodic longitudinal intensity oscillations resulting in a series of luminous areas separated by dark segments. Some scientists have related bead lightning to BL, as Boichenko[10], assuming they are both weakly ionized plasmas (with a temperature of about 5,000 K) lasting up to 1 s.

Basically, plasma consists of a quasi-neutral system of electrically charged particles, interacting among themselves and showing a collective behavior whose dynamics is mainly driven by electromagnetic forces. Although we can talk about single-species non-neutral plasmas, a plasma state usually consists of two species of charges, electrons and ions, which can also coexist at different temperatures in a nonequilibrium thermodynamic state, even in the presence of neutrals[11] or charged dust grains. The electromagnetic nature of bead lightning is commonly accepted, and, in fact, this phenomenon is most probably due to an irregular self-pinch effect in the lightning current that almost cuts itself off periodically. The pinch effect is concerned with compressional forces, due to the Lorentz forces experienced by moving charges in a magnetic field. A plasma cylinder can be confined by means of an external azimuthal magnetic field produced by surface currents along the cylinder axis. This is the pinch effect, by which a flux tube tends to contract[11]. The magnetic force $\mathbf{F}_m = q(\mathbf{v} \times \mathbf{B})$, acting on a moving charge $q$ with velocity $\mathbf{v}$ in a magnetic field $\mathbf{B}$, leads to the Lorentz force density $\mathbf{J} \times \mathbf{B}$, appearing in the magnetohydrodynamics (MHD) momentum equation

$$\frac{\partial \rho \mathbf{v}}{\partial t} + (\mathbf{v} \cdot \nabla) \rho \mathbf{v} = \rho_q \mathbf{E} - \nabla p + \mathbf{J} \times \mathbf{B} + \mathbf{F}_a . \quad (1)$$

Here $\rho$ and $\rho_q$ are the mass and charge fluid densities, **E** and **B** stand for the electric and magnetic fields, $p$ is the isotropic pressure, and $F_a$ is any additional force density (as self-gravitating force in astrophysical plasmas and viscosity or friction effects) acting on the plasma moving at velocity **v**. The Lorentz force term is directed inward in the radial direction if **B** is the azimuthal self-magnetic field of a cylindrical plasma filament, with axial current density **J**. Roughly speaking, if this current is axially modulated, this cylindrical self-pinched structure would become unstable and the plasma column can be strangled itself, leading to a series of separated beads. The irregular axial conditions in a lightning channel can be the result of several electrical and collisional effects in the column driven by wave propagation inside the lightning channel. A rigorous treatment would require the analysis of the development of channel modulation through the study of transversal and longitudinal wave propagation, as pointed out by Uman[5] and discussed in Barry[2]. As in the case of BL, there is no widely accepted explanation for the bead lightning phenomenon.

## St. Elmo's Fire

**St. Elmo's fire** is a violet or blue glow discharge observed at the end of pointed objects, such as ship masts or aircraft edges, in stormy weather conditions. Usually, these discharges keep at rest or have small displacements over a metallic wire. They can be explained as electrostatic effects of corona discharges, energetically fed by the energy associated to the atmospheric electric field. St. Elmo's fire shares most of the characteristics observed in BL; for instance, they are both spheroidal-shaped luminous objects appearing under stormy situations. Indeed, several reported BL events could be explained as special cases of St. Elmo's fire. Unlike St. Elmo's fire, however, BL can move almost freely, without remaining in contact with metals or any electrified surface. This is, probably, the most surprising characteristic reported for BL; although they follow straight motions most of the time, they also exhibit erratic motions with no appreciable change in height. Lowke has suggested that these motions could be due to the effect of superficial electric currents spread over the soil if a BL is an electric discharge, *see below*.

This suggests that the structure of BL is very probably electromagnetic and that nonsteady currents and magnetic fields inside the ball would be involved in the explanation of its properties. In this sense, the usual blue color of St. Elmo's fire, explained recently as an effect of ionized atmospheric nitrogen, can be shown by BL as well, whose richer variety in colors can be related to the ionization of several kinds of air molecules. Such ionizations could be produced by the acceleration of charges, resulting as an effect of the reconnection of magnetic lines.

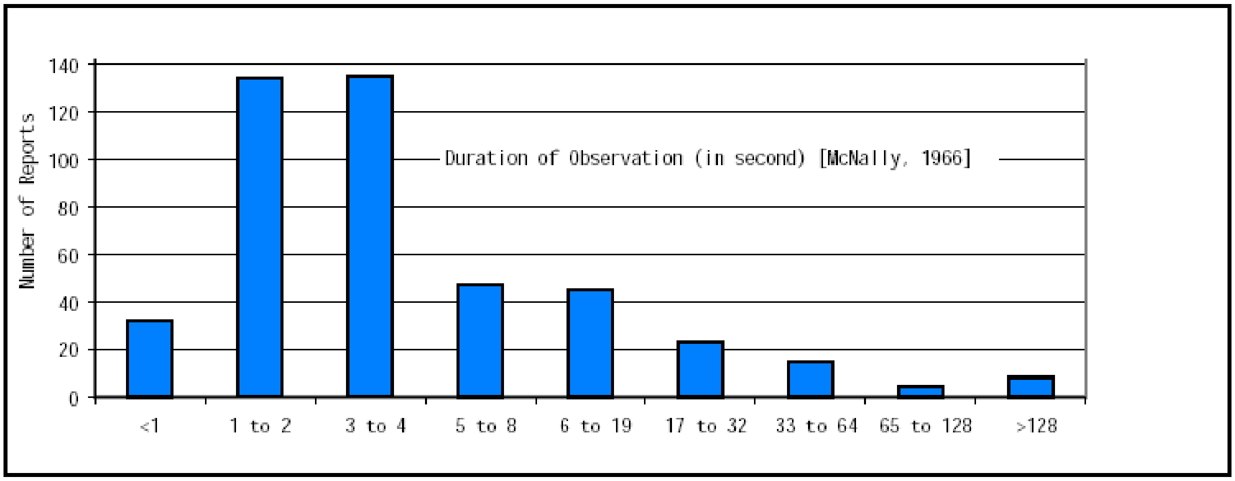

**FIGURE 3.** Distribution for time of observation, in 445 reports, from data referenced in [4]

The explanations proposed for bead lightning and St. Elmo’s fire strongly indicate that electromagnetic models of BL are the best candidates to model their properties successfully, although much work must still be done. Some researchers have looked for mechanisms that could yield the electric breakdown capable of producing a lightning ball. We must mention the effort done by Prevenslik[12] to achieve a unified theory of sprites, BL, and St. Elmo’s fire as clusters of graupel particles, using Planck´s Theory of sonoluminiscence (light emission produced by sounds) to explain the radiated light, although this is not a properly understood phenomenon.

## CLASSIFICATION OF BALL LIGHTNING AND ITS MODELS

As has been said before, there could be several different phenomena that are considered to be BL. It would be desirable to have a clear-cut classification of BL, but this is difficult because it is characterized by many different properties. Some scientists advocate the existence of several kinds of BL[4], attending to their origin and end: from cloud-to-ground or cloud-to-cloud lightning bolts; free floating or attached to conductors; coming from a lightning flash or those seen in midair, which apparently appear without nearby lightning or stormy weather. Another criterion distinguishes two types of BL according to their method of demise: either smooth noiseless dissipation or noisy explosion. In spite of these difficulties, there is a consensus on a way to classify the existing models. Finkelstein and Rubinstein[13] proposed a classification in two groups, according to whether the energy source is internal or external, i.e., according to whether the energy or the ball is stored in the system at the very beginning or it is sustained by an external feedback energy source. In the first group, there are models based on plasmoids (equilibrium configurations of plasmas), high-density plasmas with quantum mechanical properties, closed loops of currents confined by their own magnetic field (in some cases the linking of the currents play an important role), vortex structures (such as whirlwinds, rings, or rotating spheres), bubbles containing microwave radiation, chemical reactions or combustion, fractal structures, aerosols, filaments of silicon, carbon nanotubes, nuclear processes or new physics, even primordial mini black holes. In the second group, some assume that the balls are powered by electrical discharges or by high-frequency microwaves (even cosmic rays) focused from thunderclouds.

A second scheme for the classification of models relies on whether the main properties of the structure and the production of energy are of chemical or physical nature. The models of the first kind are based on chemical reactions or compounds, such as oxidation processes or polymeric structures, the chemical composition being responsible for the ball structure or formation. On the other hand, there are models that are based on electric discharges, the properties of plasmas or optical phenomena, without giving explicit emphasis to the chemistry of the balls. We can speak, therefore, of *chemical models* and *physical models*. However, new theoretical and experimental developments in physics and chemistry are entering into the task of finding an appropriate explanation of this intriguing natural phenomenon. In the following sections, we will briefly consider some of the more promising or widely discussed models, with emphasis on the most recent.

## CHEMICAL MODELS

Chemical processes have been claimed to explain BL since early times, as those based on chemical reactions or slow combustion processes of organic matter[4]. It is argued against purely chemical models that chemical reactions cannot provide the reported high energy contained in BL. However, complex chemical phenomena have been used to model BL events, as in Turner[14], who underlines the importance of electrochemical processes in this task. Among the great variety of papers reviewed in Stenhoff’s monograph, we mention here the early works of Smirnov[15].

## Ball Lightning and Chemical Geometric Structures

During the last few years, several models have been proposed as explanations for the long lifetime and the large energy stored in fireballs. A number of them base the stability on geometrical configurations, such as filamentary plasmoids, spatial structures, charged aerosols, and chemical fibers of polymeric aerogels. Some interesting models have been proposed, claiming adequate chemical compounds to form quasi-stable long-lasting skeletons as supporting BL. In 1993, Smirnov proposed that BL could be composed of low-density substances, forming knots of fractal fiber structures with a rigid skeleton. Such structures, although having the density of a gas, could be stabilized by weak distribution of charge to reach an equilibrium state. Energy is provided by chemical reactions and luminosity is due to thermal waves along the fibers, with glowing warm zones of about 2000 K; different colors are emitted by excited chemical compounds in local spotted structures. More recently, at the International Symposium of Ball Lightning in 1999 (ISBL99), Smirnov suggested that fractal structures, such as the skeleton of BL in a system of interwoven fibers, are a result of the interaction of a plasma with a solid surface.

For the last 2 years, the idea of skeleton structures of nanometer size has also been proposed by some authors who make a case for filamentary structures inside a BL. For instance, Kukushkin[16,17] proposed that the assemblage of a BL as a stable body would be achieved through an hybrid of plasma and an aerogel structure. Some kinds of these aerogel structures have been obtained under controlled conditions in the laboratory. These properties strongly suggest that BL could be a highly critical phenomenon, where metastable substances can exist in extreme conditions.

## Nanoparticles Oxidation and Network Structures

Abrahamson and Dinniss[18] proposed a network of fibers consisting of chains of particles of nanometer size. This network is formed after a lightning strike impacts on the soil. The nanoparticles could oxidize if they contain metal compounds. The glow appearance and energy release of BL would be related to these oxidation processes. To support their model, some laboratory experiments were performed[19], related to the natural production of fulgurites (glassy formations caused by lightning strikes on sand) and with the conditions to form chains of particle aggregates. The observed open-chain structures are justified using the attractive force between permanent electric dipoles under some strict conditions. Among these conditions, specified low gaseous concentrations of ions are required to avoid the screening of the local dipole, as well as mild turbulence to favor mixing in order to bring the larger agglomerates near each other[19]. In extrapolating experimental conditions into an atmospheric context, Abrahamson states a set of physical and chemical conditions to explain the formation and maintenance of a radiating BL, which can be summarized as follows:

1. Production of metal vapor by the supply of energy at high temperatures produced in electrical discharges, ohmic heating, or frictional forces in an earthquake.
2. Some carbon is also necessary, since the oxidation of this vapor “should not compete strongly with its solidification” into nanoparticles.
3. Absence of strong turbulence, flows, or shock waves in order to maintain a coherent networked structure.

These assumptions would support a theoretical model that can explain several features of BL, such as the penetration through small cracks in window panes or walls. Because of the flexibility of silicon filaments, the network carried away by the air currents to the crack could be compressed and reformed on the other side.

Taking as a basic premise the requirement of regular lightning striking an object (as soil) containing metals or oxides, a realization of the Abrahamson and Dinniss model can be explained as follows: the reaction between the silicon oxide and the carbon produces silicon metal in vapor in the heat of the lightning. After that, the silicon condensates into small nanometer spheres that are brought together resembling long fibers. If the lightning digs a small hole in the ground, serving as a very hot channel, the silicon vapor is then ejected from this cavity in the form of a vortex ring that diffuses into a spherical glowing form. The oxidation processes of the structure continue while the sphere is moving over the soil. This ball can be hot and optically visible while burning from oxidizing the silicon. A schematic graphical representation of the process can be found in Abrahamson[20].

Even if this model itself requires a strict confluence of conditions to carry out BL formation and maintenance, it seems to be plausible and well proposed. This model has two interesting features: first, the energy of the BL is explained by the emission due to the complete oxidation of silicon, and second, no plasma state is required after the ball formation to maintain the brilliance.

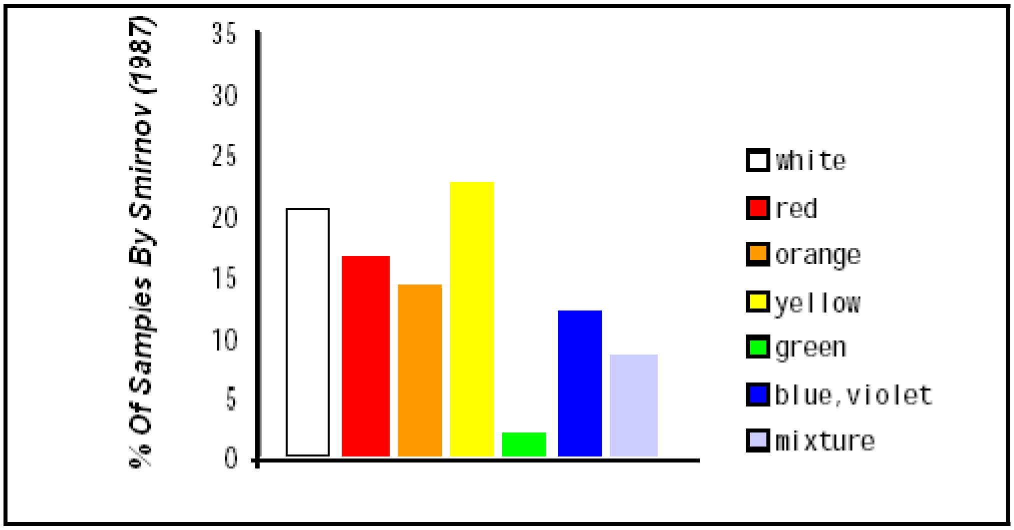

**FIGURE 4.** Percentages for the most typical BL colors, from some averaged data cited in Stenhoff[4].

## Polymeric Structures

One model that has attracted some attention states that BL can be a polymeric composite matter. In the 1990s, Bychkov proposed that BL could be composed by some kind of organic or inorganic structure similar to the form and properties of polymeric chemical compounds[21,22]. For Bychkov, a lightning strike can transform many materials in the environment into polymer fibers that can tangle up into a porous ball. The energy can be stored in the ball as a result of electric charge accumulation (under specified distribution) in the tangled dielectric polymeric structure. Heating and luminescence would arise from local breakdown discharges in the proximities of high-voltage charged surfaces. Some kind of corona discharge accompanied by internal intermittent electric currents could explain energy emissions. This chemical-physical model has been developed for more than 10 years. Thus, at the International Symposium on Ball Lightning in 2004 (ISBL04), Bychkov and coworkers extended their polymer composite model to include the possibility of high-energy storage through a unipolar charge bubble ball. The charged bubble (with melted surface) could have high electrostatic energy with density energy accumulation up to $10^3$ kJ/cm$^3$. The model of BL as a highly charged polymer-dielectric structure is the result of the aggregation of natural polymers, such as lignin and cellulose or other natural dust particles. Some experimental works on erosive discharges could support the hypothesis of these kinds of formations in a laboratory. The main problem is how to explain the fireballs coming from cloud to earth with this model.

Chemists also focus attention on the role played by dust and aerosols in the explanation of BL. These elements seem to be relevant in other phenomena, such as the production of local singularities in electric fields producing leader streamers in lightning flashes. Charged or polarized dust grains seem to drive electric transport governing the dynamics of dusty or partially ionized plasmas. As Kikuchi pointed out[23], these charged grains can induce self-organization processes for the generation of coherent electrodynamic vortices to establish complex structures. To underline the relevance of electrochemical processes and local intense fields in BL, Kikuchi proposed, at the ISBL99, a method for the reproduction of an artificial BL in cusped electric fields.

To end this section, it seems clear that the search for theoretical models of BL must be approached from a wide interdisciplinary perspective, as emphasized by Turner, for instance. In his engaging recent paper[24], Turner updated his own BL model based on chemical reactions and on the physical chemistry of ions in saturated water vapor. The model requires electric storm fields as an energy source and makes use of physical ideas. The conundrum of BL behavior is understood as the result of electrochemical processes on the surface of wet-air plasma. More precisely, the ball would operate as a “thermochemical heat pump powered by the electric field” with a central plasma core, an intermediate zone containing hydrated ions, and the exterior “refrigeration zone” providing stability through a balance of forces. For Turner, BL would be the result of an equilibrium of different effects, involving temperature and pressure gradients (due to the composition), as well as electromagnetic and gravitational fields. Several disciplines should be invoked for a proper explanation of BL, in the frame of electrochemistry.

## PHYSICAL MODELS

Many purely physical models have been proposed since the first survey of BL reports in 1838 done by French physicist Dominique F.J. Arago[4]. Most of them involve electromagnetic fields, especially after the electrical nature of lightning bolts was elucidated by Benjamin Franklin. Although some early models proposed during the 19th century are certainly obsolete if analyzed under a modern perspective, their statements and hypotheses are still being considered. This is the case, for instance, of those based on vortex structures, as well as the remark by Faraday in 1833 that BL cannot be an electrical discharge phenomenon because it would decay much faster, almost instantaneously. At the time, the nature of electrical discharges was not properly understood, but his statement is still a handicap for the elaboration of electromagnetic models of BL. As in the case of chemical models, many of the proposed hypotheses are under continuous revision and improvement. Some of them are better understood now, thanks to new ideas as self-organization or self-organized complexity. For a model considering BL as a self-organized structure, see Sanduloviciu and Lozneanu[25] for instance. In particular, geometrical considerations play an important role in both physical and chemical models.

### Kapitsa’s Model

The interest in explaining BL was much stimulated by the publication in 1955 of a paper by S. Kapitsa (cowinner of Nobel prize of Physics in 1978 for his contributions to Low Temperature Physics) proposing a model based on electromagnetic high-frequency localized discharges. Though it was built on some previously suggested ideas, the model attracted much attention, especially on how to produce BL in a laboratory. Basically, Kapitsa’s model states that an electromagnetic resonant wave is established in the atmosphere, producing a quasi-steady spherical localization of charges, under the assumption of trapped electromagnetic microwave radiation in a plasma shell that is energetically fed with external sources. He obtained a resonant condition, depending on the dimensions of the plasma ball and this justifies the absorption of radio waves in a previously weakly ionized plasma, whose ionization degree increases at the same time that volume varies, until a resonant condition relating wavelength (about 1 m) and ball diameter is reached. If the ball is heated, an expansion will destroy the resonant condition, cooling the plasma because of the reduction of the energy absorption and keeping a stable structure. The ball itself must be a set of antinodes (space points under total constructive wave interference) for an almost stationary wave. The nonconvective motion follows the antinodes at a certain constant height from the soil, where the emerging electromagnetic field causes the ball to be produced and maintained.

An attractive aspect of Kapitsa's work is its explanation of the surprising penetration indoors of BLs, because "they follow the path of short-wave electromagnetic oscillations that propagate through apertures or chimney conductor as long as a wave guide"[4]. He continued the study of BL for more than a decade, relating it to lightning radio emission and filamentary plasma structures floating in high-frequency fields. In spite of the requirement of a steady source of focalized electromagnetic wave that is not measured in the atmosphere, Kapitsa's model suggested a great number of microwave plasma experiments leading to the formation of plasmoid fireballs. One interesting example of a possible experimental realization of Kapitsa's model can be found in the work by Ohtsuki and Ofuruton[26] on the formation of spherical fireballs inside a metal cavity. Ofuruton created a shining and moving plasma ball that could pass through a ceramic plate using a 5-kW microwave generator as a 0.6-kJ capacitive discharge. In another experimental laboratory simulation of BL[27], Brandenburg and Kline produced some fireballs in a nonresonant microwave chamber. Surprisingly, the balls persisted up to 0.4 s after microwave shutoff.

From the theoretical point of view, Kapitsa's model stimulated the search for mechanisms for electromagnetic wave localization. For instance, Tanaka and Tanaka[28] proposed that BL could be an "Anderson localization". Such localization of an electromagnetic field could be achieved through constructive interference of randomized scattered waves in random media due to some sort of stochastic resonance. By numerical simulation inside a metal corridor with irregular walls, they showed that an intense localized electric discharge can be produced. Similar situations could happen in a valley, in a street, in a submarine, or in the fuselage of an airplane, as they claimed.

## Plasma Models and the Virial Theorem

Another widely discussed model was proposed by Finkelstein and Rubistein in 1964[13], when studying the possibility of plasmoidal BL. In their paper, special attention was paid to plasma models bearing charges, electrical currents, and electromagnetic fields while the matter within the ball may be either in stationary, oscillatory, or turbulent states. They established, on the basis of the magnetic virial theorem, that the confinement of a plasma system in vacuum is not possible by self-fields alone because of the conservation laws of energy and momentum. However, if there is a constant pressure outside the ball, that obstruction is removed, although there is a severe restriction to the maximum amount of stored energy; at the time, that amount was supposed to be of the order 1 MJ, two or three orders of magnitude higher than the average energy content attributed now to BL 20–30 cm in diameter. This first energy estimation was based on the quantity of water that remained warm up to 20 min after BL struck a rain barrel. Again, we now know that energy storages of 1 MJ are exceptionally rare.

The virial theorem, formulated by Chandrasekhar and Fermi in 1953[29] for astrophysical plasmas, states basically that the sum of the energies (gravitational, electromagnetic, and kinetic fluid energies) has to be null in order to achieve the system stability. In a more recent formulation applied to magnetized plasmas and using the MHD approximation, Shafranov[30] concluded that, in the absence of gravitation, bounded equilibrium configurations of astrophysical plasmas can only exist in the presence of certain current distributions. This is because, if the energy is positive, the system expands unless some forces, such as pressure differences, act inside the ball. As indicated by Stenhoff[4], Shafranov suggested that BL could be a ring structure formed from a normal lightning bolt. In any case, the question of whether a plasmoid can exist in open atmospheric air is still a subject of investigation and controversy. The restrictions imposed by the magnetic virial theorem are frequently thought to be a serious impediment for electromagnetic plasma models of BL. A simple derivation of such a theorem can be obtained by multiplying both sides of Eq. (1) by $\mathbf{r}$ and integrating over the plasma volume $V$ bounded by a surface $S$.

Using the MHD continuity equation $\partial\rho/\partial t + \nabla \cdot (\rho\mathbf{v}) = 0$, if no fluid goes out of the volume, $\mathbf{v} \cdot d\mathbf{S} = 0$, we obtain

$$\frac{1}{2}\frac{d^2 I}{dt^2} = 2T + 2U + U_B + M \qquad (2)$$

for $\mathbf{F}_a = 0$, which is a mathematical expression of the transient (time depending) virial theorem. Here, we have

$$M = (1/\mu_0)\int_S [(\mathbf{r}\cdot\mathbf{B})(\mathbf{B}\cdot d\mathbf{S}) - (B^2/2)(\mathbf{r}\cdot d\mathbf{S})] - \int_S p\mathbf{r}\cdot d\mathbf{S}$$

and

$$I = \int_V dV\rho r^2,\ T = \int_V dV\rho v^2/2,\ U = \int_V dV 3p/2\ \ U_B = \int_V dVB^2/2\mu_0.$$

$I$ is called the moment of inertia; $T$, $U$, and $U_B$ are the kinetic, internal, and magnetic energies. To explore the existence of plasma confined by its own magnetic field, the following argument is made. If the pressure $p$ is zero outside $V$, and the field decays faster than $1/r^3$, by allowing the integration surface to go to infinity, the term $U_B$ would produce a very rapid increase of $I$, i.e., an explosion, since nothing would balance the large magnetic pressure $B^2/2\mu_0$. Furthermore, in a hypothesized steady plasma state in vacuum, with the exterior pressure $p_e = 0$, with $\mathbf{F}_a = 0$, the former relation would lead to $2T + 2U + U_B = 0$, which cannot hold, proving that plasma cannot be confined by its own magnetic field in absence of external conducting walls. So, the virial theorem would exclude any electromagnetic model of BL, unless an exterior pressure $p_e$ exists. If this is the case, we have to allow the pressure to be $p_e$ at the surface $S$, to obtain

$$2T + U_B = 3(p_e - \langle p\rangle)V \qquad (3)$$

for a stationary plasma system with inner average pressure $\langle p\rangle$, lower than $p_e$. We thus infer that the maximum energy storage cannot exceed the value of $3p_eV$ if a maximum depression is allowed inside the ball, as stated by Finkelstein and Rubinstein.

The previous arguments could not apply if the system is within a bounded surface. The reason is that there might be some surface effects that are not taken into account in the preceding formulation, which only applies to average values. In some cases, the surface terms could lead to a negative contribution to the energy balance. This could happen in the presence of highly intense electric fields in localized regions, as in dusty plasmas for which $\mathbf{F}_a \neq 0$, or if the charge neutrality is locally violated ($\rho_q \neq 0$). Moreover, another important case in the above discussion is missed. If the magnetic field satisfies $\mathbf{J} \times \mathbf{B} = 0$, called "the force-free condition"[31], there is no pinch effect and all the terms involving $\mathbf{B}$ in Eq. (2) vanish, so that Eq. (3) takes the form $2T = 3(p_e - \langle p\rangle)V$, whatever $B$ is. Observe that this relation cannot be obtained as a particular case of Eq. (3), which is frequently assumed to be of general validity. If the fluid is stationary, $\mathbf{v} = 0$, a plasma could exist with no depression as it happens, for instance, in a dust or smoke halo, with the stability provided by a force-free (or Beltrami) field with high cohesive magnetic forces.

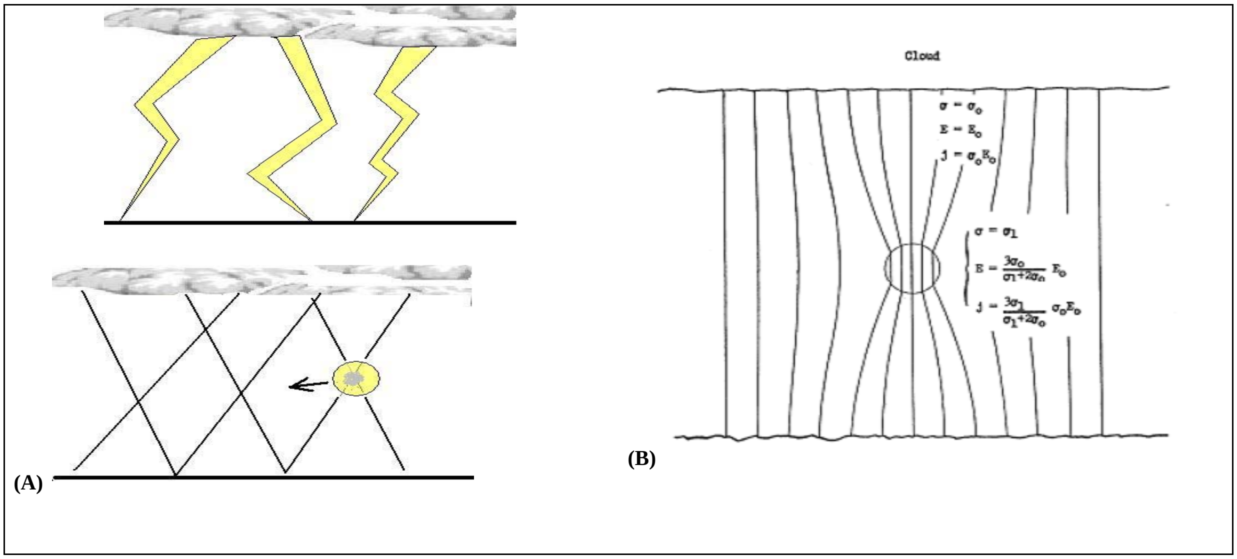

**Figure 5.** A) Scheme for Kapitsa's BL model. The ball is formed in an antinode point of electromagnetic wave interference, produced after a lightning bolt. B) Finkelstein and Rubinstein scheme of a BL as a glow discharge with non-linear conductivity σ surrounded by a Townsend discharge. Reprinted from Fig. 4, with kind permission, from D. Finkelstein and J. Rubinstein, Phys. Rev. Vol. 135, A390 (1964) . Copyright (1964) by American Physical Society.

## Finkelstein and Rubinstein Model

If Kapitsa's model stimulated the study of the experimental aspects of BL, Finkelstein and Rubinstein fostered their theoretical analysis. Short-lived plasma systems had been discovered experimentally some time before, their stability being due to a magnetic field trapped inside the system, which determines the geometry of the global structure. These systems were called plasmoids by Bostick in 1956[4]. Although Finkelstein and Rubinstein[13] found great limitations implied by the virial theorem, they proposed a possible scenario in the same paper for a plasmoidal BL as a localized discharge in a larger Townsend discharge regime (high voltage and low current).

They dealt first with the possibility of a plasma model in external atmospheric pressure, assuming that by virtue of the virial theorem, a plasma system cannot be confined by its own magnetic field if it is in vacuum, unless it is enclosed in a vessel of conducting walls. They estimated the confinement time, magnetic field, and current intensity, assuming the well-known dissipative processes in ionized media. Using typical plasma parameters to evaluate the heat flow and the electrical conductivity, they found an inconsistency between theoretical prediction for the confinement time $\tau$ and the reported lifetime of average BL. After that, they obtained a relation between the internal energy $E$ for the ball of volume $V$ and the external pressure $p_e$, using the general nonstationary formulation of the magnetic virial theorem, and assuming that the plasma inside the ball is in depression with respect to air pressure. The plasma would fill all the volume in an isotropic pressure state. The resulting energy contained in a 1-l plasmoidal ball could not exceed about 100 J (at most $3p_eV$ in a stationary state) with a lifetime of a few microseconds, as tested experimentally by Finkelstein and Powell a few years later. However, they suggested the possibility of a time-dependent plasmoid having a nonlinear conductivity depending on the current density $J$. The plasma ball was assumed to be in an infinite space within a constant asymptotic electric field at a large distance. One possible solution gave a Townsend discharge surrounding a spherical glow discharge (intermediate voltage and current), with a uniform electric field, and continued by a parallel dipole field in the Townsend region. The convergence of the electric field lines and the currents into the ball could maintain a conducting gaseous state.

Several other possibilities overcoming the restrictions predicted by the virial theorem have been suggested. Indeed, the results obtained by Finkelstein and Rubinstein do not preclude the existence of low energy plasmoids in BL. Some works have proposed MHD plasma models in order to increase the pressure difference between the ball and air. For example, Wua and Oakes[32] use a variational approach to show that the lowest inner pressure (and greater inward pressure gradients) in the atmosphere corresponds to an elongated plasma ball. It must be stressed that the virial theorem does not exclude the existence of long-living localized plasma states, as shown by Faddeev and Niemi[33,34]. Moreover, the influence of complex geometries and topological field structures, which can make the plasma state highly anisotropic, is not well described by the classical formulation of the virial theorem in a simple connected space. Thus, in our opinion, a simple formulation of the virial theorem clearly overlooks several interesting plasma configurations that can exist in Nature, as indicated by Bergström[35], who conjectured about the existence of strong charge interaction through dielectric attraction in a charged medium. In such a medium, the permittivity and the permeability would become space dependent, giving rise to an attractive Yukawa electromagnetic field in a state for which the usual virial theorem does not apply.

For the last few decades, several plasma models have been proposed based on structures and scenarios to which the virial theorem does not apply. The presence of a positively charged solid core, surrounded by a pure electron plasma layer with a magnetic field trapped inside it, was proposed by Muldrew, as referenced in Stenhoff[4]. Recently, a BL core model consisting of a cloud of electrons and ions that oscillate around each other has been proposed by Shmatov[36]. Photon emission from a highly energetic BL (1 MJ) is the probable cause of injury to human beings reported in some events. The localization of electromagnetic vortices, quantum effects, charged dust particles, and the existence of non-neutral plasma states have also been proposed[1,4]. At present, nonideal plasma states can be invoked to look for general formulations of the classical virial theorem, allowing for new theoretical frameworks for long-living plasmoids that, indeed, have been experimentally found. In these new scenarios, two of us proposed[37] an electromagnetic model formed by linked and knotted plasma streamers. Along a similar line, Witalis showed in 1990[4] that the virial theorem does not impede the existence of a magnetically self-confined two-fluid plasma. The same could happen in a two-temperature anisotropized plasma regime in a magnetic field. Both possibilities represent systems far from thermodynamic equilibrium, where the static virial theorem would not apply. We stress that further exploration of the predictions of classical MHD regarding possible plasma states is important. In this sense, we mention the BL model proposed by Kaiser and Lortz[39] in which BL is a lightning-induced plasma fireball with a decaying magnetic field at infinity. They also consider a mathematical model with spherically bounded plasma in a simple connected domain. They find that the plasma stability is improved with slight deformations of the spherical shape. Although they honestly accept that this simple model cannot explain the long lifetimes of BL, their solution in the frame of ideal MHD is worth investigation. In a similar line of research, some interesting MHD solutions modeling BL are given by Bogoyavlenskij[40].

## Ball Lightning as an Electric Discharge

Another interesting and widely discussed model was proposed by Lowke in which BL is understood as a corona discharge sustained by the electric fields associated with moving charges in the earth after a lightning strike[41]. Unlike St. Elmo’s fire, a pulsed electric field is proposed here to maintain the discharge. This externally powered model also gives an explanation of the formation, lifetime, and motion of the lightning ball. The model is enriched by several experimental and calculated data of measured electric fields and transport coefficients for realistic air plasmas. As a previous requirement, Lowke proposed that the ball be initiated by a cloud-to-ground lightning strike that transfers negative charge to the earth, while positive charge is also transferred to the cloud in a time of about 1 ms, as a return stroke. The negative charges move on the ground surface producing an electric field above the earth, responsible for the motion and power of the BL. After an initial breakdown period with a high electric field (30

kV/cm) producing air plasma, the electrical discharge is maintained by a lower electric field of about 5 kV/cm, eventually produced by the dispersed charge of 20 C moving radially from 600 m. A reduced number of electrons serves as seed to increase their density through ionization processes in high-field regions before diminishing, due to attachment to oxygen molecules and recombination with positive ions. Within the ball, the electric field driven by the continuity and the Poisson equations changes because of the charges' separation. The numerical computation shows that certain reignition processes produce pulses of currents that could not be accurately computed because of complex competitive feedback processes of temperature and ionization variability. However, these current pulses of about 1 μs could explain audible and radio frequency noises, as well as the production of ozone and nitrogen oxides in a complex ionized air. The dispersal of charges by the motion of filamentary currents on the ground would be responsible for erratic motions and the creation of a ball on the other side of a window pane. Explosive decay of the ball would happen if local electric field intensity is high enough to produce arc discharges inside the system[42]. If it is not able to reproduce all BL properties, this model has the merit of showing how environmental conditions and plasma chemical composition can be responsible for the global behavior, and how electrochemical models can be improved through the assumption of plasma scenarios.

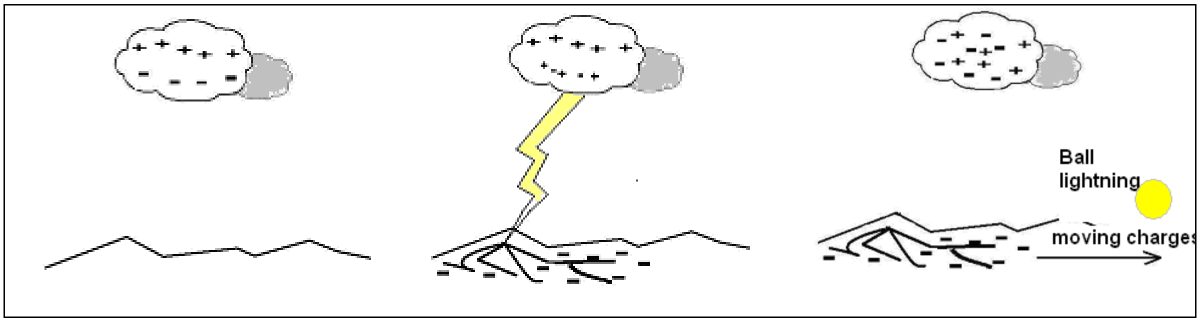

**FIGURE 6**. Scheme for Lowke's BL model. The dispersion of moving charges, after a lightning strike on the ground, creates an intense electric field that sustains BL as a corona discharge[41,42].

A model based on a very rapid, rotating electric dipole was proposed by Endean[43] with zero net current. The radial rotating electric field gives high-energy containment, overcoming the limitation of the virial theorem since, for electrostatically charged plasmoid without a magnetic field, the electric pressure at the surface is negative and the virial constraint is relaxed. This theoretical model suggests that there is no need for chemical reactive processes or structural substances.

Another purely electrostatic model can be found[44,45] where Mesenyashin modeled BL as electrostatically charged multipolar shells of water molecules, forming an ordered structure of oriented dipole moments along an electric field. Other electromagnetic models have been proposed. For instance, Natyaganov[46] considers an electrocapillary BL, understood as a cluster of spherical Hill-Taylor-like vortices, or Nikitin's model[47], based on a capacitor form of charged compressed core with a dielectric envelope, proposed at the ISBL99.

## An Optical Model

Sometimes, it is argued that the disparity on the reported BL characteristics indicates that an explanation would require new physics. Under this conviction, Torchigin[48] proposed a new BL model that, at first glance, could be classified as a purely optical one. Torchigin and Torchigin[49] explore the behavior of a hypothetical, self-confined, spherical layer of intensive light that experiences total inner reflection. The light circulates in a spherical compressed air layer that works as an optical thin-film wave-guide, after emerging from a lightning strike bolt[48]. It would propagate inside the ball, much as in optical fibers without coating, or inside glass spheres of reduced thickness. This would be possible because the refractive index would be higher than in the surrounding air, which would allow the total reflection at the two spherical surfaces of the shell. To prevent the expansion of compressed air, they believe that the intense light inside the layer produces electrostriction pressure that tends to keep close air molecules. Where BL might be a self-consistent system where compressed air confines light waves, light avoids pressure equilibrium with the air surrounding the compressed layer.

The electrostriction pressure $\Delta p$, induced by the light in a fluid, arises from the variation of the air permittivity $\varepsilon$ with the medium mass density $\rho$ as

$$\Delta p = \rho \frac{d\varepsilon}{d\rho}\frac{E^2}{2} = \frac{\rho}{\varepsilon}\frac{d\varepsilon}{d\rho}U_E \qquad (4)$$

where $E$ is the light electric field strength and $U_E$ is the energy density of such a field. The refraction index $n$, related to the vacuum permittivity $\varepsilon_0$ as $k \equiv \varepsilon/\varepsilon_0 = n^2$, can increase with gas density $\rho$ because of a lineal dependence of this magnitude with $\varepsilon_0 - \varepsilon$. Torchigin and Torchigin[50] reviewed the model and explored several scenarios that could sustain a great variation of the refraction index. They found that some of the reported properties of BL could be explained under the assumptions made in a first formulation of the model. For instance, the penetration into flying aircraft is explained assuming that BL moves along air density gradients towards the side where the density is higher. They believe that there is usually no plasma in BL after its formation as a consequence of an electric discharge, where plasma plays a fundamental role. In spite of this, they do not reject a possible double-charged layer working as a capacitor, as those found in some laboratory experiments resembling the existence of certain autonomous objects, or artificial BL.

The effect of air electrical permittivity changes is also applied in other models. For instance, Fredkin, Mayergoyz, and Zhang[51,52] applied a previous analysis of resonant dielectric objects to investigate the effect of changes of the electric permittivity as an explanation for nucleation and formation of BL. In their explanation, the permittivity becomes negative and the wavelength very large at some frequencies in resonant dielectric objects. They assume that, in the electromagnetic radiation produced by a lightning strike, there are frequencies for which the permittivity is negative in the formed plasma. This allows electrostatic resonances that produce accumulation of electromagnetic energy, visualized as a fireball. Other more exotic, but plausible, models invoke Rydberg atoms[53]. Here Gillman proposes that BL consists of highly excited Rydberg atoms (quantum state monovalence atoms) with large polarizabilities, explaining the cohesion between particles as a result of photon exchange forces, instead of using chemical forces (exchange of electrons) or magnetic field effects.

## ARTIFICIAL BALL LIGHTNING

Up to now, there have been some interesting theoretical insights, but no model of BL can explain all of its reported properties; the situation is similar in the experimental setting since, although several fireballs have been produced in laboratories, none of them is generally accepted as an experimental realization of the real thing. For a review of the most successful experiments, as those performed by Golka and Dijkhuis, among others, see for instance, Ohtsuki[3] and Stenhoff[4]. The relation between theory and experiment is crucial here; in fact, the theoretical models and the experimental works are mutually stimulating. In any case, the number of theoretical ideas and hypotheses exceeds, by far, the possibility of experimental testing, a less-than-satisfactory situation from the viewpoint of the scientific method. This is partially compensated by the enormous effort dedicated to careful study of the eye-witness reports. Nevertheless, one must never forget the goal of producing a fireball under physical conditions that can be extrapolated to those existing during the natural appearances of BL. Only a fireball showing a great correlation with field observational evidence should be called experimental BL. It can happen that some laboratory experiments, while certainly interesting on their own, may not refer to anything similar to the real BL that appears in Nature. As in the case of natural BL, several laboratory fireballs are poorly understood or remain unexplained, as the so-called spherical plasma layers. These globular formations arise in low-pressure, current-carrying plasmas. They can be observed with the naked eye, attached to biased electrodes and are, probably, composed of different plasmas in single or multiple layers[54,55].

Similar fireballs, as those reported by Alexeff[56], can be found in atmospheric air pressure. It is remarkable that these plasma spheres, several centimeters in diameter, persisted up to 0.5 s after eliminating the power source supply. The experiment shows how a plasma ball can be formed from a plasma arc, in the form of a disc, by upward convection. The plasma ball consists of a sphere of hot air confined by atmospheric pressure. It is produced slowly, to prevent supersonic shock waves, with a two-dimensional plasma source to avoid a lineal arc development. During its formation, convective losses are reduced by rotating the plasma sources; the emitted radiation (due to molecular bands in nitrogen) turns out to be negligible, if compared with thermal conduction energy losses. In previous experiments, Alexeff[57,58] found sets of closed-current rings, first accidentally observed in high-voltage sparks, claiming that they were precursors of BL. The self-confining magnetic field plays an important role in these structures. The high-energy content seems to contradict some estimations of the virial theorem.

Some experimental works have investigated the influence of internal magnetic fields in the formation of fireballs, most of them being related to plasma confinement devices. Koloc[59] argued that natural BL can be a stable plasma configuration, sharing several characteristics with spheromak plasmas. Alas, these are only found in the laboratory under controlled conditions in low-pressure vacuum chambers surrounded by metallic walls. However, similar cold-fluid plasma structures have been found to persist after the suppression of external magnetic field, as in Chen et al.[60]. Here Chen, Pakter, and Seward found, both theoretically and experimentally, the equilibria of a class of stable self-organized electron spiral toroids that could explain toroidal BL.

On the other hand, some theoretical chemical models have motivated the work on fireballs obtained from electrical discharges that drag chemical compounds or metallic ions (called erosive discharges). For instance, Avramenko et al.[4,61] studied erosive discharges and obtained plasma states with unusual properties (luminescence, spherical shape, and 1-s lifetime) that are similar to those reported for BL. Moreover, the effect of metallic residuals in the lifetime of a plasmoid is also of great interest. Experiments of this kind are also motivated by the observation of 1-s green fireballs in submarine batteries, probably related to electrode erosion in accidental short circuiting. Dijkhuis[62], performing experiments with a submarine battery and 150-kA currents, produced and filmed fireballs with a diameter of 10 cm and a lifetime of about 1 s. He suggested that the phenomenon is due to some kind of quantum-mechanical exchange force. More recently, Shavanov[63] obtained reproducible, luminous globular formations (about 10 cm in diameter, lasting up to 1 s) that appear in erosive discharges having typical BL colors. If the balls are touched with a metal wire, they eject some substance. This property suggests the existence of a core and a shell in the objects, which might be composed of two interpenetrating plasma structures. Special attention is devoted here to the optical properties and the influence of the observational conditions to describe the ball colors. Sometimes, it is observed that the same ball, classified as belonging to the “short-living” group, can exhibit several colors, with a lifetime of less than 1 s. In Shavanov’s opinion, this result seems to confirm the existence of two types of BL. The optical behavior (even transparent) under flash illumination, when photographed, is quite different to that observed without the flash. Both observations are related to background colors and color perception by humans, a fact that has to be taken into account in the witnesses’ reports.

An analogous set of experiments has been performed by Emelin and coworkers, another Moscow research group[64,65], using high-voltage erosive discharges between ring-shaped anode and a dry rod cathode, both immersed in water.

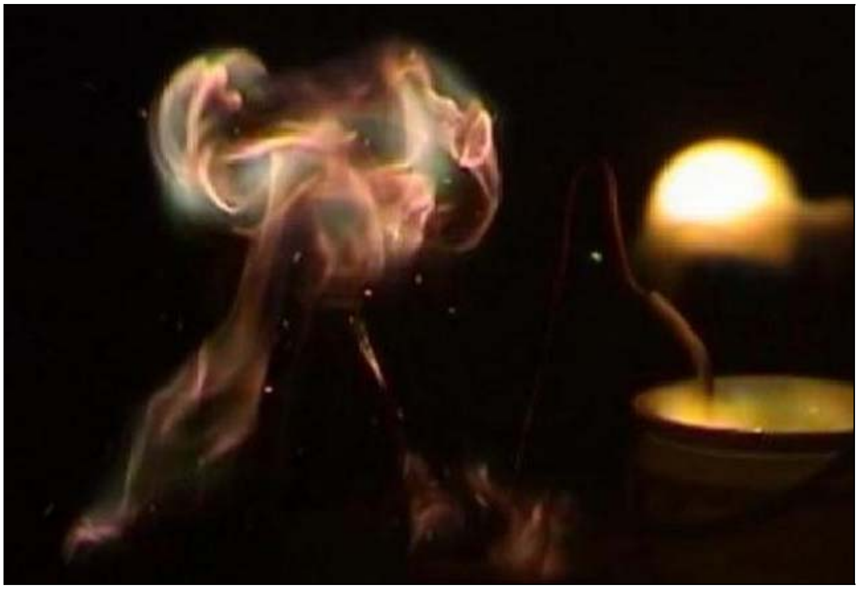

**FIGURE 7.** Two experimental fireballs in an erosive discharge by Emelin et al., one showing the streamers, the other a shell. From http://balllightning.narod.ru/hvewd.html, with kind permission.

The 10- to 20-cm luminous spheres are apparently surrounded by a thin-film elastic envelope enclosing gas and, sometimes, they have a filamentary structure, as shown in Fig. 7. Emelin et al. believe that the dispersion of metal and polymers inside a small volume during the discharge forms an almost critical mode, leading to an active medium with high stored energy. A set of self-organization processes results in the autonomous objects. However, without claiming the existence of complex polymeric structures, Egorov and Stepanov[66] carried out similar experiments, establishing the atmospheric conditions required for natural BL formation: high electrical activity and water vapor. They argue that the dipole water molecules attack free ions and surround dust or aerosol particles. They believe that hydrated ions (positive and negative) come close together maintaining additional water molecules to form clusters that give rise to spatial structures. The previous chaining would prevent recombination of charges in a plasmoid. The initial energy in the discharge accumulated upon formation of ion pairs and persisted for an extended period of time in the cold plasmoid (330 K in temperature).

## RECENT PLASMA MODELS

It is argued, sometimes, that a plasma model of BL in atmospheric conditions is unreliable because it would disappear in a very short time due to energy emission and recombination of charges with no external sources. This assertion is usually related to the analysis of the paradigmatic plasma state found in short-living lightning or in fusion devices, such as tokamaks or stellarators. Thus, it is sometimes forgotten that the so-called “fourth state of matter” is really quite a variety of states of different properties. They go from fully ionized, high-density hot plasmas to supercooled metastable plasmas (with a high degree of ionization even at low temperature) and astrophysical, almost collisionless, plasmas at a very low density. Moreover, in atmospheric conditions, the role played by charged dust particles may configure a complex (dusty) plasma state that could give rise to new plasma scenarios with some of the properties reported for BL. Recent research has revealed an ability to generate a huge amount of self-organized structures, comprising crystal and liquid plasmas by means of the presence of charged dust particles. This new theoretical framework could tell us much about BL.

Some of the early models dealing explicitly with this new physics on dusty (complex) plasma theory can be found in Tsytovich[67] and Smirnov[68], implying geometrical structures conditioned by the existence of micrometer- or nanometer-charged grains. In this context, and because of the electrical nature of many atmospheric phenomena, it is reasonable to look for a plasma model of BL, especially because a lightning discharge is also a plasma system. Obviously, there are many obstacles when building a plasma model. The main problem in the construction of a coherent plasma model is how to explain what kind of particle cohesion could lead to a long time confinement. Several mechanisms have been proposed to explain plasma cohesion, but the simplest way to achieve it is with a magnetic field. As in fusion devices, the problem of plasma confinement, thus, is intrinsically related to the structure of the applied and self-generated magnetic field. To confine plasma, one must confine the magnetic lines and vice versa, as happens in stellarators and tokamaks. Some pure electrical models have been proposed to overcome the problem of particle cohesion and better explain other effects, as a way to reduce recombination of charges that would lead to the plasma extinction. Inclusion of a magnetic field is essential, not only for plasma cohesion. Moreover, if a pure electric field is invoked, the fireball penetration in an aircraft, for instance, could not be explained since a plane is a Faraday cage, an impenetrable wall for the ball. On the other hand, a magnetic field could “pass” a metallic or dielectric wall, a fact that would allow the reformation of the ball in the “other” side.

## Force-Free Models

In addition to models that assert some kind of nuclear reactions, or fusion-related processes, the existence of atmospheric plasmoids is treated in some new models, in spite of the constraints of the virial theorem. Tsui[69] considered BL as a self-organized phenomenon with plasma immersed in a spherical force-free magnetic field. This field, completely aligned with the plasma current everywhere, implies that no pinch force is exerted on the plasma as discussed in previous sections. An experimental recreation of this system, among others, is found in a spheromak plasma that spontaneously evolves to a force-free state from given initial conditions. Since the field does not exert force on the former, due to the zero Lorentz force density, Tsui assumes the plasma to be in mechanical equilibrium with the surrounding air. After a mathematical analysis of the possible field solutions, Tsui does not reject the existence of highly energetic singular magnetic fields, whose strength is only truncated by the limitations of the possible physical currents. Large energy storage could be a consequence of self-organization of the magnetic and current vortices. A deeper discussion of this model is presented in his more recent and interesting paper[70]. In it, Tsui shows that the force-free magnetic field configurations, such as high-energy atmospheric plasmoids, are not subjected to the constraints of the virial theorem. The reason is that the plasma is pressure balanced by the atmosphere, not by the magnetic field itself. The absence of an electric field implies that there is no dissipation of the magnetostatic equilibrium. The system topology plays a fundamental role because of the self-preservation of magnetic helicity $h$ a term first coined by Moffatt[71,72,73,74], defined as the volume integral

$$h = \int \mathbf{A} \cdot \mathbf{B} \, dV \quad (5)$$

where $\mathbf{A}$ is the magnetic vector potential. The helicity is a topological property of the field configuration, whose constancy forces the system to decay through a cascade of force-free states. This would provide a lifetime of several seconds. The force-free plasma configuration has been proposed as well by Callebaut[75] at the last Symposium on Ball Lightning (ISBL 2004), as a possible initial phase of a large class of BL.

## A Topological Model

Recent research on self-organization and filamentation processes in plasmas has aroused great attention. The natural tendency of plasma to self-organize into filamentary or layered structures can be observed in several scenarios in Nature and in the laboratory. Using these essential features of plasma, a topological model describes BL as a system of linked or knotted streamers that may occur in a lightning discharge as a result of self-organization processes. The formation of the BL is still a matter of investigation. However, it is frequently admitted that it may result from a complex set of plasma instabilities in the discharge column of a lightning bolt. The topological model uses the concept of streamer as a channel of traveling electric charges with filamentary (neutral) plasma structures, carrying currents. In this way, our streamers may differ substantially to arcs, mainly if they may be closed or ring currents loops, possibly formed by charge or current collapse of a previously evolutionary, trapped closed current. In this model, the stability is due to the topological configuration of a set of streamers under linked magnetic lines without the recourse of solid structures. In fact, as Trubnikov said[76], the usual theoretical tools can be used to explain filamentation processes in plasmas, such as the closed current loops discovered recently[77]. Moreover, some theoretical works have found that some MHD solutions embody polymeric-like localized plasma filamentary structures[78].

A plasma model of BL should take into account three remarks:

1. The power emitted by plasma of the size of BL is too high (1 l of air plasma at 15,000 K emits about 5 MW, several orders of magnitude too much.) This may be an indication that most of the ball is at ambient temperature, only a small fraction being hot, concentrated in filamentary structures as hot current streamers. If this fraction is of the order of 1 ppm, the radiated output would be of the order of 10–100 W, in agreement with the witnesses' reports.
2. It can be argued that any plasma current channel inside the ball would be necked and cut in a very short time by the pinch effect. However, there is no pinch effect if $\mathbf{J} \times \mathbf{B} = 0$, which is equivalent to $\nabla \times \mathbf{B} = \lambda\mathbf{B}$. These force-free magnetic fields inside plasma correspond to minimum energy relaxed states, as shown by Chandrasekhar and Woltjer[79].
3. A third remark is based on the magnetic virial theorem, which states that a system of charges in electromagnetic interactions has no equilibrium state in the absence of external forces because the large magnetic pressure must produce an explosion with no other force to compensate it. However, in principle, the fireballs are not in equilibrium, but in metastable states with slow evolution. Still more important, the force-free condition annihilates the magnetic pressure or, at least, reduces it to a much smaller value if the field is just almost force free.

The fact that BL may contain force-free magnetic configurations of plasmas is plausible. Since electric conduction in air proceeds through thin channels called streamers, as it happens in ordinary lightning, it can be imagined that plasma inside the fireball consists of a self-organized set of metastable, highly conductive, wire-like or filamentary currents. Furthermore, unusual long-living filamentary (even closed loops[58]), high-density structures have been theoretically predicted and experimentally observed. Filamentary states with minimum dissipation in a magnetic field, with twisted force-free interacting current channels, were found by Gekelman and coworkers[80]. Here the authors stress the role played by the self-generated magnetic fields in a dynamic driven by electron pressure gradients, which increase the helicity as the channels twist about each other. In the theoretical frame, the problem needs a much more complex analysis than what has been done up to now; for instance, one must include very important considerations, such as the thermochemical and quantum effects on the transport processes in the plasma as well as other nonlinear effects. Faddeev and Niemi[33] proposed, in 2000, compelling arguments that challenge certain widely held views on plasmas. They showed that the virial theorem does allow nontrivial equilibrium states of streamers and electromagnetic fields inside a background of plasma, which are "topologically stable solitons that describe knotted and linked flux tubes of helical magnetic fields", as had been proposed by Rañada et al. in 1998[37,38].

The topological model[81,82,83] describes BL as two systems in interaction: (1) a magnetic field with its magnetic lines linked to one another and (2) a set of linked streamers containing a plasma of ionized air. The model assumes a plasma of ionized air confined inside some closed streamers, carrying electric current flows and a magnetic field with linked lines coupled to the streamers. In Fig. 8, a representation of the streamers as currents flowing inside a fireball in the topological model is seen. In this case, any two of the six streamers shown are linked once. The hot air plasma is confined in the set of streamers, its relative volume being small, and the rest of the ball is at ambient temperature.

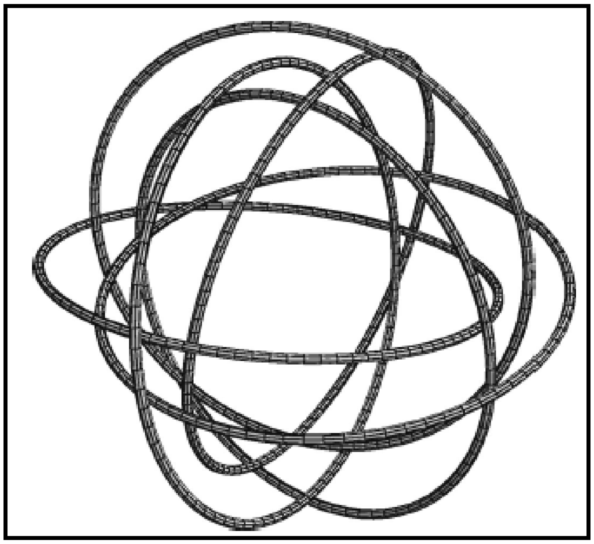

**FIGURE 8**. Schematic representation of the magnetic lines in the topological model of BL. This magnetic knot was built from the Hopf map. The linking number of any two lines is 1.

This model uses the concepts of magnetic knot and magnetic helicity $h$, Eq. (5). A magnetic knot is a magnetic field with finite energy such that (1) its force lines are closed, the level curves being of some complex scalar field, and (2) the linking number $n$ of these force lines is a constant of the motion and it does not depend on the pair of lines (so that the magnetic field has topological properties). If a magnetic knot **B** appears in the free space, then its magnetic helicity can be written as $h = na$, where $a$ is a constant and $n$ is the linking number of the magnetic lines. In this BL model, we have a resistive plasma coupled to a magnetic knot. We use the momentum Eq. (1), coupled to the induction equation $\partial \mathbf{B}/\partial t = \nabla \times (\mathbf{v} \times \mathbf{B})$, both in MHD single-fluid approximation. If $\eta$ is the resistivity of the plasma and **J** the electric current density, the magnetic helicity is conserved if $\eta \mathbf{J} = 0$. This condition holds approximately inside the streamers, where $\eta$ is very small, while outside $\mathbf{J} = 0$. In other words, the magnetic field is nearly a magnetic knot, with topological property that stabilizes the structure.

The model distinguishes three stages. The first stage of BL is its formation. As it happens in ordinary lightning bolts, the air does not conduct as a continuous medium, but along thin tubes of highly conducting plasma called streamers (about 100 μm in diameter in a lightning bolt). Near an ordinary lightning discharge, some streamers can short circuit, forming closed and linked loops. The model assumes that the force-free condition $\mathbf{J} \times \mathbf{B} = 0$ holds, so that stream lines and magnetic lines coincide, having the same topology: the magnetic lines are also closed and linked.

In a second stage, the fireball suffers a very rapid Taylor relaxation[84,85] in which the magnetic field lines break and reconnect, without changing the helicity, producing heating and charge acceleration. Note, after that, there are no mechanical losses of energy, since the power density $\mathbf{v} \cdot \mathbf{J} \times \mathbf{B}$ is zero. Ohmic losses are also minimized for very weak resistivity along field lines. The relaxed state is a force-free magnetic knot, with the initial value of the helicity, which cannot dissipate energy any further. If the initial helicity is nonvanishing, then the structure is very tangled with the plasma fluid moving along the magnetic lines. Consequently, the magnetic lines and the current lines can have the same linking number *n* and this marks time zero in BL.

The third stage is the BL phase. We could have a stationary state after the Taylor relaxation if we neglect the radiation and resistive losses. Strictly speaking, the resulting open system cannot be in equilibrium because its energy decreases as the radius of the ball increases, radiating this extra energy. But if the helicity is almost conserved, this decay is very low, it would be an almost quiescent state, and that explains the long lifetime. Eventually, the ball cools and the conductivity decreases, which leads to the structure destruction[81].

Although this model was formulated in the ideal MHD, further research must include a stability analysis and dissipative effects. About the stability, we can say that global stability of several plasma structures can be ensured for some relaxed states involving force-free fields[86]. About dissipative effects, a deeper study should have to include the effect of strong magnetic fields that tend to anisotropize a system governed by two different dynamics, parallel and transversal to the magnetic field. A wide class of anisotropic equilibria may be derived from ideal MHD solutions, preserving the topology of the system[87,88]. Each of these solutions may be a candidate to describe BL plasma equilibrium. A two-temperature plasma, in which dissipative processes can be drastically reduced in the transversal field direction, arises naturally while the conductivity may be very high along the field[89]. A deeper theoretical analysis would require dealing with kinetic theory in plasmas to evaluate the transport coefficients under high electromagnetic fields and temperature gradients[90]. The effect of the magnetic field inhibition of both thermal conduction and charge diffusion would also be responsible for the long lifetime of BL, since the associated transversal transport coefficients decrease with the magnetic field strength as a function of $1/B$.

The reader might find that the quantity and variety of BL models are confusing. However, BL is not the only natural phenomenon in which some controversy has occurred. For example, it is well known that streamers reported in early stages of atmospheric discharges can split into branches spontaneously, but how this branching is determined by the underlying physics has been a matter of discussion[91,92,93].

## SUMMARY

Ball lightning is an amazing phenomenon still unexplained after nearly 2 centuries of scientific efforts to elucidate its nature. On the one hand, it poses an unavoidable challenge to human curiosity and pride. On the other, the attempts to find an explanation of its properties offer interesting insights into the behavior of a number of physical systems. It is usually accepted that unexplained phenomena can be found only in the realms of the very large, the very small, or the very complex phenomena. The fireballs are neither large nor small, in fact, they are in the mesoworld and may not seem to be very complex. However, the experience gathered thus far suggests that they have, most probably, a really complex structure, which touches poorly explored regions of Nature, as is the case for many unexplained effects found in ordinary lightning.

The surprising and intriguing properties of the balls do not seem easy to explain with just a simple idea. Indeed, there is no lack of theoretical proposals, although none is generally accepted as the solution to the riddle; on the experimental side, the generation of fireballs in the laboratory has shown to be a difficult task. This has led to some confusion, with some scientists claiming that there are various kinds of balls under the same heading, explainable in the future through diverse physical or chemical mechanisms and requiring different approaches and strategies.

There are chemical and physical models. The former models attribute the stability of the balls to the chemistry of some particular compounds, the latter models base their structure on electromagnetism or perhaps nuclear energy. There are compelling arguments that point to an electromagnetic structure of the balls, although they collide with some interpretations of the magnetic virial theorem. In fact, nobody other than Faraday himself stated that a fireball cannot be an electromagnetic phenomenon, since it would explode in a split second. His remark has had a certain negative influence on electromagnetic models. However, we know now that the electromagnetic systems of plasmas, currents, corona discharges, electromagnetic fields, and waves can be very complex and that their analysis is much more difficult than what was assumed at first sight. Furthermore, the conclusions of the virial theorem need some implicit assumptions and cannot be valid where there are microscopic effects; for instance, where the wave functions can be considered as a macroscopic field (see Faddeev and Niemi[33]).

Some conclusions or suggestions can be drawn from this review.

1. A good theoretical model for the fireballs should: (1) explain their long lifetime, (2) provide a mechanism for the confinement of their structure, (3) elucidate the source of their energy and whether it is external or internal, and (4) show why they tend to move horizontally with some chaotic bouts.
2. Before resorting to exotic theories, one should explore the standard well-known chemistry or physics, as applied to new structures not considered before. In this sense, new approaches on self-organization processes could be relevant when constructing an appropriate model.
3. On the experimental side, the only laboratory phenomena that should be considered to be genuine BL should have similar properties to those reported for the naturally appearing balls, especially long life and luminosity. It is particularly important that they be produced under conditions that can be extrapolated, under scaling laws, to the atmospheric environment of natural BL.
4. It is important to “capture” BL events, i.e., observe them on the spot, where they are reported to be frequent, as well as the surrounding environment. This would allow us to take pictures and films with filters and still cameras, analyze the spectra of their radiation, and measure their size and lifetime, the electromagnetic fields, the environmental conditions, etc. This is not easy, since it would be necessary to install an outdoor laboratory for continuous observations for a long period of time, in distant and not easily accessible places.
5. The research on BL concerns several disciplines and has necessarily some degree of interdisciplinarity. Its general framework is electrochemistry. One must not forget in this sense that chemical effects play a role in plasma physics, for instance, in the new fields of dusty plasmas. Moreover, although BL is a macroscopic phenomenon, quantum effects may be important, since they are always implied in plasma physics.

## ACKNOWLEDGMENTS

This research has been partially supported by the Spanish Ministry of Science and Technology, under the project grant BFM2003-05453.

## BIOSKETCHES

**José M. Donoso** lectures in the Department of Applied Physics at the Polytechnic University (ETSI Aeronáuticos) in Madrid, Spain. He obtained his PhD at the Complutense University of Madrid on numerical integral methods for Fokker-Planck Plasma Physics equation. At the moment, he studies nonlinear kinetic effects in plasma transport processes involving electromagnetic fields and numerical computation on nonlinear plasma equations.

**José L. Trueba** is a lecturer in the Applied Physics Department, Universidad Rey Juan Carlos, Móstoles, Spain. He studied physics at Universidad Autónoma of Madrid and received his PhD at Complutense University of Madrid on topological configurations in classical electromagnetism, including a model of ball lightning. His main interests are electromagnetic knots, plasma physics, and waves in excitable media.

**Antonio Fernández-Rañada** is Professor of Physics at the Complutense University of Madrid, Spain, since 1976. After working several years on elementary particle physics following his doctorate at Paris University, his career interests have been quantum physics and nonlinear dynamics, then topological fields, proposing a model of electromagnetism in which some quantities as the charge and the energy in a cavity obey quantization rules due to the topological configuration of the field lines. This inspired a model of ball lightning, in which the linking of magnetic lines increases the stability of the fireballs. He works now in cosmology, where he proposed a model for the Pioneer Anomaly. He founded and directed the journal *Revista Española de Física* for 10 years and was a member of the Council for Ethics of Scientific and Technical Research of the Spanish Science Foundation. He is presently the president of the Royal Spanish Physical Society.
